# Supplementary material for: Antitumor Efficacy of Doxorubicin-Loaded Electrospun Attapulgite–Poly(lactic-co-glycolic acid) Composite Nanofibers
Source: J Funct Biomater. 2022 May 10;13(2):55. doi: 10.3390/jfb13020055 (PMC9149849; doi:10.3390/jfb13020055)
Supplement: Supplementary file 1 [file jfb-13-00055-s001.zip › jfb-1659280-supplementary.pdf]

Antitumor efficacy of doxorubicin-loaded electrospun attapulgite–poly(lactic-co-glycolic acid) composite nanofibers

Zhe Wang [1,2], Yili Zhao [3], Mingwu Shen [2], Helena Tomás [4], Benqing Zhou [1,*], Xiangyang Shi [2,4,*]

[1] Department of Biomedical Engineering, College of Engineering, Shantou University, Shantou 515063, China

[2] Shanghai Engineering Research Center of Nano-Biomaterials and Regenerative Medicine, College of Chemistry, Chemical Engineering and Biotechnology, Donghua University, Shanghai 201620, China

[3] College of Textile Science and Engineering (International Institute of Silk), Zhejiang Sci-Tech University, Hangzhou 310018, China

[4] CQM-Centro de Química da Madeira, Universidade da Madeira, Campus da Penteada, 9000-390 Funchal, Portugal

---

* Corresponding author. E-mail addresses: bqzhou@stu.edu.cn (B. Zhou) and xshi@dhu.edu.cn (X. Shi)

Part of Experimental Details:

Materials

PLGA (molecular weight = 81000 g/mol) with a lactic acid/glycolic acid ratio of 50: 50 was purchased from Jinan Daigang Biotechnology Co., Ltd. (Jinan, China). DOX was purchased from Beijing Huafeng Pharmaceutical Co., Ltd. (Beijing, China). ATT was from Mingguang Jianxi Dongfeng Mine Products Factory (Mingguang, China). Dimethylsulfoxide (DMSO), tetrahydrofuran (THF), N,N-dimethyl formamide (DMF), RPMI 1640 medium, fetal bovine serum (FBS), phosphate buffer saline (PBS), penicillin, and streptomycin were purchased from Gibco (Carlsbad, CA). Resazurin was from Sigma-Aldrich (St Louis, MO). A human osteosarcoma cell line (CAL72) was from the University of Madeira (Funchal, Portugal). Water used in all experiments was purified using a Milli-Q Plus 185 water purification system (Millipore, Bedford, MA) with a resistivity higher than 18 MΩ·cm.

***In vitro* drug release**

The release kinetics of DOX were determined by measuring the DOX absorbance at 490 nm using a UV-vis spectrophotometer. The ATT/DOX complex (5 mg) was dispersed in 1 mL of phosphate buffer saline (PBS, pH = 7.4) or sodium acetate–acetic acid buffer solution (pH = 5.4). The dispersed solution was then transferred to a dialysis tube, which was placed in a vial containing 10 mL of the corresponding buffer solution. Similarly, PLGA/DOX and ATT/DOX/PLGA nanofibers with the same DOX concentration were directly placed into different vials containing 10 mL of the corresponding buffer solution. All the samples were incubated in a vapor-bathing constant temperature vibrator with a shaking speed of 90 rpm at 37 ^{o}C for a period of 10 days. At each predetermined time point, 1 mL of outerphase solution was removed from each vial for quantitative analysis using UV-vis spectroscopy. An equal volume of fresh corresponding buffer solution was replenished to the vial to keep the outer phase volume constant.

Characterization techniques

The ATT/DOX/PLGA composite nanofibers were characterized using different techniques.

For Fourier transform infrared spectroscopy (FTIR), we used potassium bromide pressed-disk technique to prepare samples; we took 2-3 mg of sample (free ATT, DOX, or the ATT/DOX particles) and 200-300 mg of dry KBr powder, mixed them in agate mortar, ground them fully, and pressed the sample to disk before measurement according to the manufacturer’s protocol. The FTIR spectra were performed using a Nicolet

Nexus 670 FTIR spectrometer over a wavenumber range of 500 to 4000 cm^{-1} to confirm the loading of DOX onto the ATT particles. The crystalline structures of ATT before and after modifications were analyzed by a Rigaku D/max-2550 PC X-ray diffraction (XRD) system (Rigaku Co., Tokyo, Japan) with a wavelength of 0.154 nm at 40 kV and 200 mA. The scan was performed from 5° to 70°. Lastly, the DOX, ATT, and ATT/DOX solutions were characterized using UV-vis spectroscopy (Perkin Elmer Lambda 25, Waltham, MA) at a wavelength range of 200-800 nm. The morphology of the ATT/DOX nanohybrid was characterized by transmission electron microscopy (TEM, JZM-2100, Japan) at an operating voltage of 200 kV. Aqueous dilute suspensions of ATT and ATT/DOX (5 μL) were dropped onto a carbon-coated copper grid and air dried before TEM measurements. For the PLGA nanofibers and ATT/DOX/PLGA composite nanofibers, the fiber sample was directly electrospun onto the carbon-coated copper grid and vacuum dried before TEM imaging. The morphologies of PLGA, ATT/PLGA, PLGA/DOX, and ATT/DOX/PLGA nanofibers were also observed by SEM (JEOL JSM-5600LV, Tokyo, Japan) at a voltage of 15 kV. All samples were sputter coated with gold films with a thickness of 10 nm before SEM observation. The diameters of the electrospun fibers were analyzed using ImageJ 1.53k software (http://rsb.info.nih.gov/ij/download.html, National Institutes of Health, USA). At least 200 nanofibers from different images were analyzed for each sample to obtain the diameter distribution histogram. The porosity, mechanical properties, and surface hydrophilicity of the nanofibers were measured according to protocols described in our previous work [1-3].

Statistical analysis

One way ANOVA statistical analysis was performed to compare the antitumor efficacy of DOX in different formulations with PBS as a negative control. A value of 0.05 was selected as the significance level, and the data were indicated with (*) for $p< 0.05$, (**) for $p < 0.01$, and (***) for $p < 0.001$, respectively.

References

[1] S.G. Wang, R. Castro, X. An, C.L. Song, Y. Luo, M.W. Shen, H. Tomás, M.F. Zhu, X.Y. Shi, Electrospun laponite-doped poly (lactic-co-glycolic acid) nanofibers for osteogenic differentiation of human mesenchymal stem cells, J. Mater. Chem. 22(44) (2012) 23357-23367.
[2] F.Y. Zheng, S.G. Wang, M.W. Shen, M.F. Zhu, X.Y. Shi, Antitumor efficacy of doxorubicin-loaded electrospun nano-hydroxyapatite–poly (lactic-co-glycolic acid) composite nanofibers, Polym. Chem. 4(4) (2013) 933-941.
[3] F.Y. Zheng, S.G. Wang, S.H. Wen, M.W. Shen, M.F. Zhu, X.Y. Shi, Characterization and antibacterial activity of amoxicillin-loaded electrospun nano-hydroxyapatite/poly (lactic-co-glycolic acid) composite nanofibers, Biomaterials 34(4) (2013) 1402-1412.

Table S1. Optimization of ATT concentrations for DOX encapsulation (the DOX concentration was fixed at 1.0 mg/mL).

**ATT (mg/mL)**	**2.5**	**3.75**	**5**	**6.25**	**7.5**	**8.75**
Loading efficiency (%)	30.3 ± 0.63	46.8 ± 0.76	62.4 ± 0.33	81.1 ± 0.25	97.2 ± 0.06	97.0 ± 0.18

Table S2. Diffraction angle and plane spacing data of ATT and ATT/DOX using XRD analysis.

**diffraction plane (*hkl*)**	**2θ peak position (deg)**		**plane spacing (*d*, Å)**	
	ATT	ATT/DOX	ATT	ATT/DOX
(110)	8.340	8.340	10.59	10.59
(200)	13.70	13.58	6.459	6.515
(040)	19.88	19.86	4.463	4.467
(400)	27.64	27.58	3.225	3.232
(102)	34.32	34.28	2.611	2.614

Table S3. Apparent density, porosity and water contact angles of PLGA, ATT/PLGA, and ATT/DOX/PLGA nanofibers.

**Sample**	**Apparent density (g/cm^3)**	**Porosity (%)**	**Water contact angle (°)**
PLGA	0.20 ± 0.075	84.0 ± 4.0	136.8 ± 2.6
ATT/PLGA	0.21 ± 0.023	75.2 ± 1.84	131.9 ± 2.7
ATT/DOX/PLGA	0.22 ± 0.037	73.8 ± 2.96	129.1 ± 1.5

Table S4. Tensile properties of electrospun PLGA nanofibers, ATT/PLGA and ATT/DOX PLGA nanofibers.

**Sample**	**Breaking strength (MPa)**	**Failure strain (%)**	**Young's modulus (MPa)**
PLGA	4.3 ± 0.79	234.7 ± 25.5	130.3 ± 11.4
ATT/PLGA	6.7 ± 1.06	154.3 ± 17.3	201.8 ± 18.3
ATT/DOX PLGA	6.5 ± 0.95	151.6 ± 21.1	223.5 ± 17.6

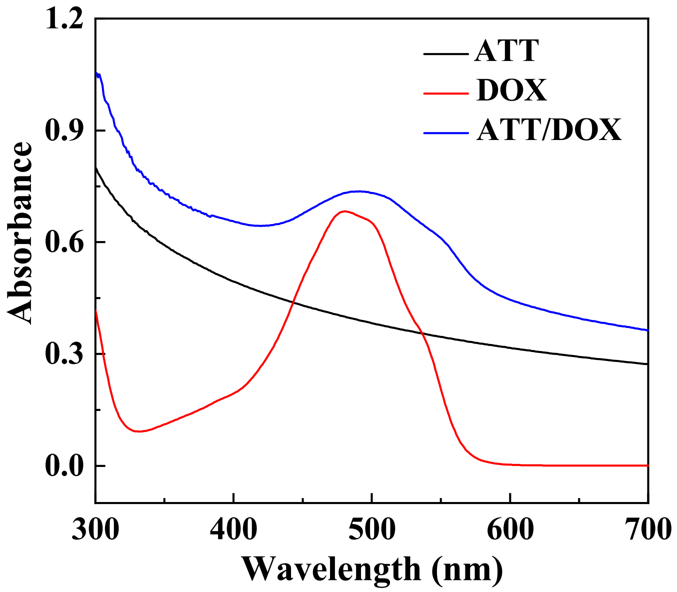

Figure S1. UV−vis spectroscopy of DOX, ATT, and ATT/DOX complexes.

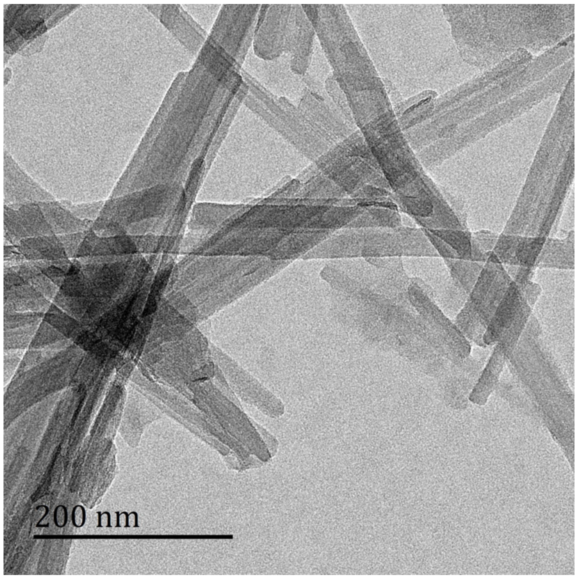

Figure S2. TEM image of free ATT.

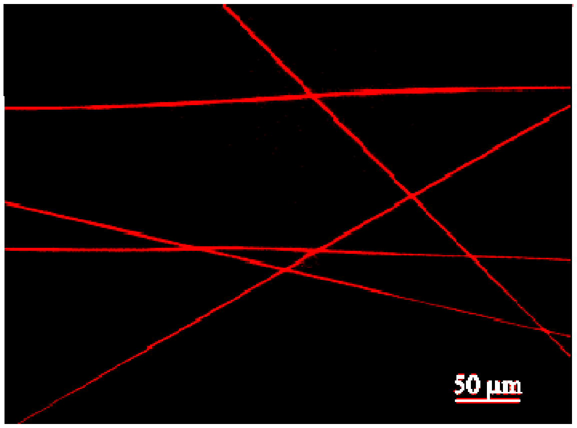

Figure S3. A fluorescence microscopic image of the DOX/PLGA composite nanofibers.

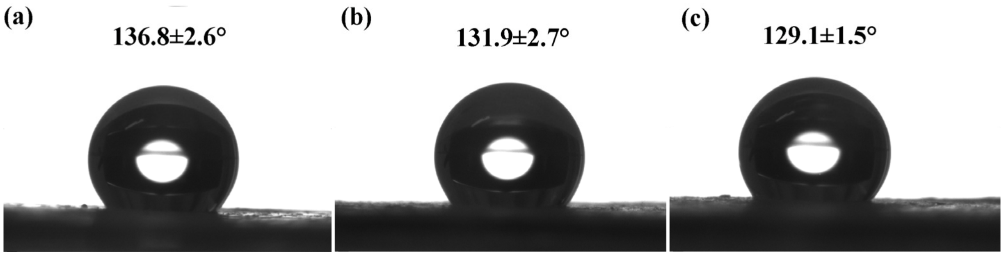

Figure S4. Water contact angles of PLGA (**a**), ATT/PLGA (**b**), ATT/DOX/PLGA (**c**) nanofibers.
